# Contribution of Emotional and Motivational Neurocircuitry to Cue-Signaled Active Avoidance Learning

**DOI:** 10.3389/fnbeh.2014.00372

**Published:** 2014-10-27

**Authors:** Anton Ilango, Jason Shumake, Wolfram Wetzel, Frank W. Ohl

**Affiliations:** ^1^Leibniz Institute for Neurobiology, Magdeburg, Germany; ^2^Department of Psychology, The University of Texas, Austin, USA; ^3^Institute of Biology, University of Magdeburg, Magdeburg, Germany; ^4^Center for Behavioral Brain Sciences (CBBS), Magdeburg, Germany

**Keywords:** punishment, active avoidance, safety signal, neural circuits, dopamine, amygdala, striatum

## Introduction

Many animal and human subjects can learn to avoid punishment or noxious stimuli by exploiting the sensory cues predicting them. In cue-signaled active avoidance (AA) learning, subjects first learn about the predictive properties of cues and subsequently learn a behavioral strategy of an avoidance response (e.g., crossing a hurdle that divides a two-compartment cage in order to avoid a mild but unpleasant footshock). AA learning develops with fear reduction as an intervening variable (Mowrer and Lamoreaux, 1946; Miller, 1948). If execution of avoidance action occurs on the pursuit of seeking safety, an overlap of recruitments of neurocircuitry essential for reward processing and for avoidance can be expected on the basis of two-process theory. Recent insights from two-way AA (2WAA) studies, which integrate both Pavlovian and instrumental components, provide strong evidence for the recruitment of emotional circuitry centered on the amygdala and motivational circuitry centered around midbrain dopaminergic structures. In this review, we address the following: (1) the role of emotional neurocircuitry in the formation of AA, (2) the involvement of reward circuitry and its input-output pathways on AA, and (3) the possible serial and parallel processing within and between these circuitries.

## Role of Emotional Circuitry in AA

Pavlovian learning increases synaptic plasticity in amygdala neurons (Tye et al., 2008) that respond to cues predicting either appetitive or aversive outcomes (Paton et al., 2006; Tye and Janak, 2007). While the amygdala has been studied extensively for its involvement in fear processing (LeDoux, 2000), recent studies highlight the amygdala as a key substrate during acquisition of AA. Studies indicate that different subnuclei of the amygdala contribute differently to the acquisition of an avoidance strategy and to the consolidation of avoidance memories. Bilateral electrolytic lesions of the basolateral amygdala (BLA) (Segura-Torres et al., 2010) and pre-training infusion of an NMDA antagonist into the BLA impair the acquisition of 2WAA learning (Savonenko et al., 2003). Central amygdala (CeA) lesions disrupt the acquisition of an AA response but have no effect on the retrieval of a previously acquired AA response (Roozendaal et al., 1993). However, for animals that failed to acquire 2WAA after 3 days of training, CeA lesions actually improved AA learning (Choi et al., 2010). Thus, the BLA appears important for all phases of AA learning whereas the role of the CeA appears to be limited to acquisition and complex, potentially facilitating or impeding AA learning depending on the specific AA response, and/or innate individual differences in AA learning ability.

During the initial stages of AA acquisition, the expression of conditioned freezing to the CS can interfere with AA learning. In such situations, the infralimbic prefrontal cortex (IL) exerts feed forward inhibition of the amygdala to reduce the expression of freezing responses to the CS while sparing the predictive association between the CS and US, which is necessary for AA learning (Moscarello and LeDoux, 2013). Studies on rabbits trained to induce wheel rotation to avoid shock during CS^+^ presentations confirm the involvement of amygdala (LA, B, and Ce), cingulate cortex, thalamus, and auditory cortex in the acquisition and retention of AA (Smith et al., 2001). Intra-BLA infusion of muscimol significantly affected the acquisition of discriminative avoidance with two tones but did not affect the CR to both CS after overtraining (Poremba and Gabriel, 1997, 1999).

## Role of Motivational Circuitry in AA

Given the heterogeneity of the ventral tegmental area (VTA), it is not surprising that dopamine (DA) neurons play different roles ranging from signaling reward and mediating motivation to coding aversion, salience, uncertainty, and novelty (Horvitz, 2000; Bromberg-Martin et al., 2010; Ilango et al., 2012; Lammel et al., 2014). Additional complexity became evident after the discovery of subsets of DA neurons co-transmitting glutamate in the nucleus accumbens (NAc) shell (Stuber et al., 2010), GABA in the dorsal striatum (Tritsch et al., 2012) and GABA in the lateral habenula (LHb) (Stamatakis et al., 2013) raising several specific questions about the involvement of this system.

Here, we will discuss the tonic and phasic DA release associated with signaled AA. Briefly, DA antagonists impair AA responses, and electrical stimulation of reward circuitry facilitates AA [see reviews by Salamone (1994) and Ilango et al. (2012)]. NAc DA release increases during the first training block of 2WAA and progressively decreases in the second training block as the number of AA responses increase (Dombrowski et al., 2013). Signaled AA learning progresses with the increase of tonic DA release in medial prefrontal cortex (mPFC) and reaches its peak during the formation of the first successful avoidance trails (Stark et al., 1999, 2001). Elevated DA release in mPFC was also found in a training scenario in which two different cues were first associated with the same meaning (signaling Go response) and were subsequently associated with different meanings (Go and NoGo) (Stark et al., 2004).

Recent studies implicate the relevance of the phasic DA signal to punishment prediction and avoidance. During the safety period, in both avoidance and escape, DA release in the NAc was increased (Oleson et al., 2012). Furthermore, brief electrical stimulation applied to the LHb contingent to the AA response (i.e., at the initiation of the safety period) impaired acquisition but not retention of 2WAA (Shumake et al., 2010; Ilango et al., 2013; Shumake and Gonzalez-Lima, 2013). Studies utilizing viral gene therapy in DA deficient mice showed that shock escape and learning 2WAA require DA signaling in both the amygdala and striatum. And after overtraining (which is more resistant to extinction), DA signaling in the striatum alone was sufficient to maintain 2WAA. In contrast, restoring DA signaling in the PFC and amygdala was insufficient to maintain AA (Darvas et al., 2011). Careful lesion experiments in different regions of the striatum confirmed that NAc core and dorsolateral striatum (DLS) lesions delayed 2WAA acquisition without disrupting the ability to acquire AA. In contrast, dorsomedial striatum (DMS) lesion did not affect the early phase but decreased 2WAA after six training sessions (Wendler et al., 2014). Pre-training infusion of D1 or D2 antagonist into the DLS did not affect the number of AA responses during training but significantly decreased AA responses during the retention test 24 h later (Boschen et al., 2011; Wietzikoski et al., 2012).

Also, the laterodorsal tegmental nucleus (LDTg) and pedunculopontine tegmental nucleus (PPTg), which send glutamatergic and cholinergic projections to the midbrain, play an important role in 2WAA (Mena-Segovia et al., 2008; Lammel et al., 2012). Bilateral lesions of the PPTg completely abolished the acquisition of 2WAA (Fujimoto et al., 1989). Rats with ipsilateral disconnection of the SNc from the PPTg learned the 2WAA, but contralateral disconnection blocked learning even after 3 days of conditioning, suggesting that PPTg-SNc communication is necessary to acquire 2WAA (Bortolanza et al., 2010).

## Perspectives

The majority of DA neurons are inhibited by aversive USs, or by CSs signaling aversive USs [for details see Ilango et al. (2012)]. Hypothetically, once the aversive CS–US association is repeated several times, the tonic inhibition mode changes and the change in DA dynamics prepare the organism to perform successful avoidance responses. Perhaps, this learning relevant change in DA signaling also led to change in synaptic plasticity occurring between hippocampal → amygdala neurons and the neurons of the direct pathway that are active at the same time, thus guiding the organism to repeat the instrumental response. There are several pathways that could provide midbrain DA neurons with information about aversive events. Nociceptive signals from the spinal cord pass pain-related signals to the parabrachial nucleus (PBN). Through its direct glutamatergic projection or indirect glutamatergic route to the rostromedial tegmental nucleus (RMTg) and VTA GABA neurons, the aversive signal is relayed to midbrain VTA/SNc DA neurons. Inactivation of the PBN either reduces the amplitude or completely abolishes the inhibitory response of DA neurons to footshock (Coizet et al., 2010). In addition, modulation of pain and aversion-related signals conveyed by the LHb reaches DA neurons directly or indirectly through the RMTg (Jhou et al., 2009). This informative signal is highly processed and capable of assigning motivational valence based on prior events. Indeed, unexpected footshock increased LHb-to-RMTg glutamate release, and optogenetic activation (60 Hz) of this pathway promoted place aversion and AA learning to prevent the activation (Stamatakis and Stuber, 2012).

There is also evidence that CeA–DA interactions may be important for AA learning. The CeA projects to substantia nigra DA neurons and to the DLS. Moreover, electrical stimulation of the CE modulates the firing of SNc DA neurons (Rouillard and Freeman, 1995). The CE is also known to strengthen the effect of Pavlovian stimuli on instrumental performance, and the CeA → DLS pathway is implicated in habit acquisition (Corbit and Balleine, 2005; Lázaro-Muñoz et al., 2010; Lingawi and Balleine, 2012). Reciprocally, the lateral part of SNc DA neurons project to the CeA, playing a role in surprise-induced enhancement of attention and learning (Lee et al., 2006, 2008). Accordingly, the first successful avoidance trial in an AA paradigm likely constitutes a “pleasant surprise” for the animal – a violation of the expectation that the CS is always followed by the US – and we hypothesize that early AA success may recruit the SNc → CeA pathway and consequently enhance attention and learning of the AA response.

**Figure 1:**
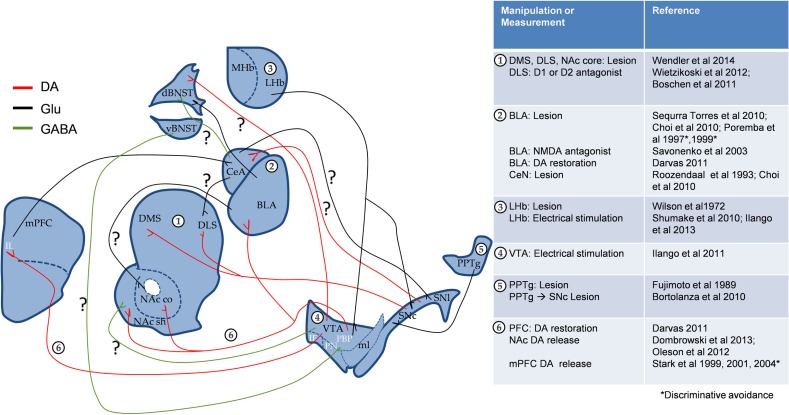
**Proposed interaction of emotional and motivational neurocircuitry in cue-signaled AA learning**. Apart from the known role of BLA DA in AA, both circuits could interact through direct SNc_←_^→^CeA projections and/or indirectly through striatum and BNST. “?” Denotes the pathways, which could potentially play a role in AA. mPFC, medial prefrontal cortex (IL, infra limbic); NAc, nucleus accumbens (c, core, and sh, shell); VTA, ventral tegmental area (PBP, parabrachial pigmented nucleus; PN, paranigral nucleus; IF, interfascicular nucleus); SN, substantia nigra (c, compact part and l, lateral part); ml, medial lemniscus; CeA, central nucleus of the amygdala; BLA, basolateral amygdala; BNST, bed nucleus of the stria terminalis; MHb and LHb, medial and lateral habenula; PPTg, pedunculopontine tegmental nucleus; DMS, dorsomedial striatum; DLS, dorsolateral striatum. To simplify the circuit, the direct and indirect pathways of striatum were excluded.

From the above-mentioned evidences, it is clear that parallel streams of information might reach the amygdala and DA midbrain structures, and both systems might interact at the striatum level (Figure 1). Blocking transmission of glutamate signals by knocking out the NMDA receptor in medium spiny neurons of the striatum impaired learning of a simple FR1 operant task for food reward as well as 2WAA. This confirms that 2WAA recruits simple motor coordination circuits (Beutler et al., 2011). Moreover, striatal specific deletion of adenosine A(2A) receptors impaired 2WAA. This is interesting given the known role of these receptors in fine-tuning the DA-glutamate balance in the striatum (Singer et al., 2013).

Unlike reward learning, when a subject masters AA, it no longer receives external motivation in the form of a US, providing a mystery to early learning theorists. How can behavior be sustained in the absence of reinforcement? One solution is that a fear memory of the US continues to be evoked by the CS, and alleviation of this fear state can continue to motivate the AA response in the absence of the US. But if the CS is no longer paired with the US, why does the CS–US association not undergo extinction? Moreover, animals that have mastered an AA task no longer show strong physiological signs of fear or distress. For these animals, the execution of the avoidance response takes on the quality of a habit. Therefore, we propose that AA learning ultimately recruits and depends on the same circuitry involved in habit formation, such as the so-called spiraling loop of striatal–nigral–striatal circuitry (Yin and Knowlton, 2006; Belin and Everitt, 2008; Ilango et al., 2014). We believe that this circuitry is a prime target for investigating the neural mechanisms that sustain avoidance behavior, and it may reveal novel ways of facilitating its extinction.

## Conflict of Interest Statement

The authors declare that the research was conducted in the absence of any commercial or financial relationships that could be construed as a potential conflict of interest.
